# Mycophenolate Improves Brain–Gut Axis Inducing Remodeling of Gut Microbiota in DOCA-Salt Hypertensive Rats

**DOI:** 10.3390/antiox9121199

**Published:** 2020-11-28

**Authors:** Iñaki Robles-Vera, Néstor de la Visitación, Manuel Sánchez, Manuel Gómez-Guzmán, Rosario Jiménez, Javier Moleón, Cristina González-Correa, Miguel Romero, Tao Yang, Mohan K. Raizada, Marta Toral, Juan Duarte

**Affiliations:** 1 Department of Pharmacology, School of Pharmacy and Center for Biomedical Research (CIBM), University of Granada, 18071 Granada, Spain; irv1991@correo.ugr.es (I.R.-V.); nestorvp@correo.ugr.es (N.d.l.V.); manuelsanchezsantos@ugr.es (M.S.); mgguzman@ugr.es (M.G.-G.); rjmoleon@ugr.es (R.J.); javiermm95@gmail.com (J.M.); cristinagoncor@gmail.com (C.G.-C.); 2 Instituto de Investigación Biosanitaria de Granada, ibs.GRANADA, 18001 Granada, Spain; 3 Ciber de Enfermedades Cardiovasculares (CIBERCV), 18011 Granada, Spain; marta.toral@cnic.es; 4 Microbiome Consortium and Center for Hypertension and Precision Medicine, Department of Physiology and Pharmacology, University of Toledo College of Medicine and Life Sciences, Toledo, OH 43537, USA; Tao.Yang2@utoledo.edu; 5 Department of Physiology and Functional Genomics, University of Florida, Gainesville, FL 32611, USA; mraizada@ufl.edu; 6 Gene Regulation in Cardiovascular Remodeling and Inflammation Group, Centro Nacional de Investigaciones Cardiovasculares (CNIC), 28001 Madrid, Spain

**Keywords:** mycophenolate, gut dysbiosis, hypertension, oxidative stress, inflammation, (deoxycorticosterone acetate) DOCA-salt model

## Abstract

Microbiota is involved in the host blood pressure (BP) regulation. The immunosuppressive drug mofetil mycophenolate (MMF) ameliorates hypertension. The present study analyzed whether MMF improves dysbiosis in mineralocorticoid-induced hypertension. Male Wistar rats were assigned to three groups: untreated (CTR), deoxycorticosterone acetate (DOCA)-salt, and DOCA treated with MMF for 4 weeks. MMF treatment reduced systolic BP, improved endothelial dysfunction, and reduced oxidative stress and inflammation in aorta. A clear separation in the gut bacterial community between CTR and DOCA groups was found, whereas the cluster belonging to DOCA-MMF group was found to be intermixed. No changes were found at the phylum level among all experimental groups. MMF restored the elevation in lactate-producing bacteria found in DOCA-salt joined to an increase in the acetate-producing bacteria. MMF restored the percentage of anaerobic bacteria in the DOCA-salt group to values similar to control rats. The improvement of gut dysbiosis was associated with an enhanced colonic integrity and a decreased sympathetic drive in the gut. MMF inhibited neuroinflammation in the paraventricular nuclei in the hypothalamus. This study demonstrates for the first time that MMF reduces gut dysbiosis in DOCA-salt hypertension models. This effect seems to be related to its capacity to improve gut integrity due to reduced sympathetic drive in the gut associated with reduced brain neuroinflammation.

## 1. Introduction

Systemic arterial hypertension is a complex, multifactorial, and multisystem disorder influenced by genetic and environmental factors, and is the most important modifiable risk factor that contributes significantly to worldwide cardiovascular morbidity and mortality. A link between gut microbiota to hypertension in both animal models and human hypertension has been described. Recently, an imbalance in the gut microbiota composition relative to the healthy state, termed dysbiosis, has been associated with hypertension [1,2,3,4]. The characteristics of dysbiosis are different between systemic renin–angiotensin system (RAS)-dependent hypertension, such as in spontaneous hypertensive rats (SHR) [1,3,5,6,7], and systemic RAS-independent forms, such as hypertension induced by mineralocorticoid receptor activation [8,9]. Studies using fecal microbiota transplantation procedures have demonstrated that gut microbiota from hypertensive animals and human increased blood pressure (BP), showing a cause–effect relationship [3,10,11], albeit the mechanisms involved in BP regulation by the microbiota have not been fully elucidated. 

Emerging evidence indicates that immune system dysfunction is an important factor in the pathogenesis of hypertension [12]. In fact, T cell activation is involved in the development of hypertension induced by angiotensin II infusion and by deoxycorticosterone acetate (DOCA)-salt [13]. The microbiome plays a critical role in the induction, maturation, and maintenance of the host immune system. Interestingly, inhibition of T cell activation and helper T (Th)17 differentiation reduced the hypertensive effect induced by dysbiotic microbiota from SHR, showing that immune system dysregulation induced by the gut microbiota may be, at least partially, responsible for the development of hypertension [3]. Interestingly, the improvement of gut dysbiosis induced by probiotic bacteria [7,9,14], dietary fiber [8], or by drug treatment [6,15], in several experimental models of hypertension, was involved in their antihypertensive effects. However, the antihypertensive drug hydralazine was unable to improve gut dysbiosis in SHR despite intensive BP reduction, showing that gut microbiota composition was not adapted to the host health status of normotension [6]. Recently, Raizada’s group has linked hypothalamic neuroinflammation and increased sympathetic drive with changes in gut physiology and microbiota associated with angiotensin II-induced hypertension [16]. This suggests a role for a dysfunctional autonomic nervous system in gut dysbiosis. 

Mycophenolate mofetil (MMF) is a prodrug of mycophenolic acid (MPA), an inhibitor of inosine 5’-monophosphate dehydrogenase. This drug depletes proliferating B and T lymphocytes and macrophages due to its ability to rate-limit enzymes in de novo synthesis of guanosine nucleotides. MMF was able to prevent the development of hypertension in DOCA-salt due to pharmacological inhibition of B and T cell proliferation [17,18]. Recently, it has been described that MMF influences the gut microbiome in renal transplant patients [19,20,21,22] and rodents [23]. Renal transplant recipients suffer from dysbiosis, characterized by lower diversity and loss of butyrate-producing bacteria, more than one year post-transplantation, and the use of MMF correlates to a lower diversity [21]. In addition, MMF exposure promoted expansion of the phylum Proteobacteria. This increase was accompanied by gene enrichment for multiple bacterial enzymes involved in the biosynthesis of lipopolysaccharide (LPS) and increased plasma levels of LPS [19]. Thus, lower diversity in gut microbiota and higher plasma LPS levels induced by MMF could be harmful under hypertensive conditions [1,3,7]. By contrast, the reduction in blood pressure and gut sympathetic tone induced by MMF could improve dysbiosis linked to hypertension. However, it is unknown as to whether the modulation of the immune system by MMF improves the remodeling of gut microbiota under mineralocorticoid-induced hypertensive conditions. Thus, the aim of this study was to analyze the effects of MMF in the gut microbiota in this model of hypertension, focusing on the involvement of the sympathetic nervous system. 

## 2. Materials and Methods 

### 2.1. Animals and Experimental Groups

The experiments performed in this study followed the European Union regulations and requirements concerning the protection of animals used for scientific purposes. Experimental protocols were approved by the Ethics Committee of Laboratory Animals of the University of Granada (Spain; permit number 03-CEEA-OH-2013). Animal studies are reported in compliance with the ARRIVE guidelines [24]. Male Wistar rats (290–350 g) were obtained from Envigo Laboratories (Barcelona, Spain). Rats were randomly divided into three groups: untreated (CTR, 1 mL of tap water once daily by oral gavage, *n =* 7), DOCA-salt (DOCA, 1 mL of water once daily by oral gavage, *n =* 8), and DOCA treated with MMF (DOCA-MMF, 20 mg/kg/day by oral gavage, *n =* 9). DOCA-salt hypertension was induced by intramuscularly DOCA administration at a dose of 12.5 mg/0.5 mL saline solution per rat and week for 4 weeks in uninephrectomized rats, as was previously described [25]. A sham operation was performed on the control counterparts and received a weekly saline injection. During the experimental period, DOCA-salt rats had free access to water containing 1% NaCl. Then, hypertensive DOCA-salt rats were treated with vehicle or MMF for another 4 weeks. The MMF solution was prepared daily and administered through oral gavage. Rats were kept in specific pathogen-free facilities at University of Granada Biological Services Unit. All animals were housed under standard laboratory conditions (12 h light/dark cycle, temperature 21–22 °C, 50–70% humidity). Rats were housed in Makrolom cages (Ehret, Emmerdingen, Germany), with dust-free laboratory bedding and enrichment. In order to avoid horizontal transmission of the microbiota among animals, we housed each rat in a separate cage. Rats were provided with water and standard laboratory diet (SAFE A04, Augy, France) ad libitum. Water was changed every day, and both water and food intake were recorded daily for all groups. Body weight was measured every week. The MMF treatment was stopped 2 days before the end of the experiments in order to study its long-term effects without the involvement of acute administration effects.

### 2.2. Blood Pressure Measurements

A 2-week adaptation period for vehicle administration and systolic blood pressure (SBP) measurements was established prior to the initiation of experimental procedures. SBP was measured weekly at room temperature using tail-cuff plethysmography, as described previously [6].

### 2.3. Cardiac and Renal Weight Indices

After the experimental period, the animals fasted for 18 h and were anesthetized with 2.5 mL/kg equitensin (intraperitoneal.). Blood was collected from the abdominal aorta. Arterial blood samples (0.2 mL) were drawn to measure noradrenaline (NA) levels. Ethylenediaminetetraacetic acid (EDTA) 1 mM and sodium metabisulfite 4 mM were added to prevent the catecholamine degradation. Then, the animals were killed by complete exsanguination. Blood samples are cooled in ice and centrifuged at 3500 rpm at 4 °C for 10 min; then, the plasma samples were stored at −80 °C for later use. The heart was taken out and weighed, and then divided into the left ventricle and the right ventricle plus atria. All tissue samples were frozen in liquid nitrogen and then stored at −80 °C.

### 2.4. Plasma Determinations

Following the manufacturer’s instructions, we used the Amebocyte Lysate (LAL) Chromogenic Endotoxin Quantitative Kit (Lonza, Valais, Switzerland) to measure the plasma LPS concentration. We used enzyme-linked immunosorbent assay kits (IBL International, Hamburg, Germany) to measure plasma NA concentrations, following the manufacturer’s protocol. 

### 2.5. Vascular Reactivity Studies

Thoracic aortic rings (3 mm) were dissected and mounted in organ chambers filled with Krebs solution (composition in mmol/L: NaCl 118, KCl 4.75, NaHCO_3_ 25, MgSO_4_ 1.2, CaCl_2_ 2, KH_2_PO_4_ 1.2, and glucose 11), as previously described [6]. The concentration–relaxation response curves to acetylcholine (10^−9^–10^−5^ mol/L) were studied in aorta pre-contracted by phenylephrine (1 μmol/L). The concentration–relaxation response curves to nitroprusside (10^−9^–10^−6^ mol/L) were performed in the dark in aortic rings without endothelium pre-contracted by 1 μmol/L phenylephrine. In some rings, acetylcholine responses were studied after incubation with N^G^-nitro-L-arginine methyl ester (L-NAME, a non-selective competitive inhibitor of nitric oxide synthase, 10^−4^ mol/L) or the nicotinamide adenine dinucleotide phosphate (NADPH) oxidase inhibitor VAS2870 (5 μmol/L) for 30 min. Relaxant responses were expressed as a percentage of precontraction.

### 2.6. NADPH Oxidase Activity

As previously described [26], a lucigenin-enhanced chemiluminescence assay was used to determine the NADPH oxidase activity in the intact aortic ring. The aortic rings from all experimental groups were incubated in a physiological salt solution (pH 7.4) containing 4-(2-hydroxyethyl)-1-piperazineethanesulfonic acid (HEPES) with the following composition (in mmol/L) at 37 °C for 30 min: NaCl 119, HEPES 20, KCl 4.6, MgSO_4_ 1, Na_2_HPO_4_ 0.15, KH_2_PO_4_ 0.4, NaHCO_3_ 1.0, CaCl_2_ 1.2, and glucose 5.5. We added NADPH (100 μmol/L) to the buffer containing the aortic ring, and automatically injected lucigenin (5 μmol/L). NADPH oxidase activity was determined by measuring luminescence over 200 s in a scintillation counter (Lumat LB 9507, Berthold, Germany) in 5-s intervals and was calculated by subtracting the basal values from those in the presence of NADPH and expressed as RLU (relative light units)/min per milligram of tissue for aortic rings.

The NADPH oxidase activity in the paraventricular nucleus (PVN) homogenate was measured by the dihydroethidium (DHE) fluorescence assay in the microplate reader [27]. A solution of fresh homogenate (10 µg protein) with DHE (10 µM) and deoxyribonucleic acid (DNA, 1.25 µg/mL) in phosphate buffered saline (PBS) 100 mM, pH 7.4, containing 100 µM diethylenetriamine-pentaacetic acid (DTPA) salt and NADPH (50 µM) to a final volume of 120 µL was incubated for 30 min at 37 °C in the dark, in the absence or in the presence of the non-selective NADPH oxidase inhibitor apocynin (50 μM). The total fluorescence was tracked in a fluorescence spectrophotometer (Fluorostart, BMG Labtechnologies, Offenburg, Germany) using a rhodamine filter (excitation 490 nm, emission 590 nm) in a microplate reader.

### 2.7. Measurement of Ex Vivo Vascular Reactive Oxygen Species (ROS) Levels

We used DHE, an oxidative fluorescent dye, to localize ROS in aortic segments in situ, as previously described [26]. Briefly, the aorta segments were included in optimum cutting temperature compound medium (Tissue-Tek; Sakura Finetechnical, Tokyo, Japan), quickly frozen, and cut into 10 μm thick sections in a cryostat (Microm International Model HM500 OM). Sections were incubated at room temperature for 30 min with 10 μmol/L DHE in the dark, counterstained with the nuclear stain 4,6-diamidino-2-phenylindole dichlorohydrate (DAPI, 300 nmol/L), and in the following 24 h examined on a fluorescence microscope (Leica DM IRB, Wetzlar, Germany). Sections were photographed and ethidium and DAPI fluorescence were quantified using ImageJ (version 1.32j, National Institutes of Health (NIH), http://rsb.info.nih/ij/). ROS production was estimated from the ratio of ethidium/DAPI fluorescence.

### 2.8. Lymphocyte-Conditioned Media Preparation

Mesenteric lymph nodes (MLN) were obtained from rats to measure lymphocyte population by RT-PCR and cytokines in lymphocyte conditioned media. MLN was carefully mashed with wet slides to reduce friction. The cell suspension obtained was then filtered through a 70 µm cell strainer. With reference to a previous study [28], 5μg/mL concanavalin A was used in Roswell Park Memorial Institute (RPMI) 1640 medium with 10% fetal bovine serum and 2 mmol/L glutamine to stimulate total lymphocytes in lymph nodes (2.5 × 10^6^/mL) and soaked in 48 mL of 10 mmol/L 6-HEPES, 100 U/mL penicillin, 100 g/mL streptomycin and 50 μmol/L β-mercaptoethanol in a 25 mL flask for 48 h. After centrifugation, the lymphocyte-conditioned medium was stored at −80 °C until use. We measured the levels of interleukin (IL)-10 and IL-17a in lymphocyte-conditioned media by ELISA kit (IL-17a, ElabscienceBiotechnology, Houston, TE, USA; IL-10, Invitrogen Life Technologies, Carlsbad, CA, USA), according to the instructions of the manufacturer and using an ELISA reader (FLUOstar optimaV1.20-0, BMG Labtechnologies, Offenburg, Germany).

### 2.9. Gene Expression Analysis

The analysis of gene expression in aorta, colon, brain PVN, or mesenteric lymph nodes (MNL) was performed by RT-PCR, as previously described [27]. For this purpose, total RNA was extracted by homogenization using TRI Reagent, following the manufacturer’s protocol. All RNA samples were quantified with the Thermo Scientific NanoDropTM 2000 Spectrophotometer (Thermo Fisher Scientific, Inc., Waltham, MA, USA), and 2 μg of RNA was reverse-transcribed using oligo(dT) primers (Promega, Southampton, UK). Polymerase chain reaction was performed with a Techne Techgene thermocycler (Techne, Cambridge, UK). The sequences of the sense and antisense primers used for amplification are described in Table S1. The efficiency of the PCR reaction was determined using a dilution series of standard vascular samples. To normalize mRNA expression, we used the expression of the housekeeping gene β-actin. The mRNA relative quantification was calculated using the ∆∆Ct method. 

### 2.10. 16S rDNA V4-V5 Region Sequencing and Bioinformatics Analysis

Fecal DNA was extracted from the samples collected from all experimental groups by using a quick-DNA fecal/soil microbe kit (Zymoresearch, Irvine, CA, USA). Primers compatible with illumina Miseq v2 2 × 250bp kit (Illumina, San Diego, CA, USA) were used to amplify bacterial 16S V4-V5 variable regions [1]. The PCR amplicons were purified by QIAquick gel extraction kit (QIAGEN, Hilden, Germany) and quantified by Qubit (Thermo Fisher Scientific, Waltham, MA, USA). Equal amounts of purified PCR product from each sample were pooled together as one library. The library was quantified by real-time PCR (Kapa Biosystems, Wilmington, MA, USA) prior to Miseq sequencing (Illumina, San Diego, CA, USA). The sequencing data had a Q30 score ≥93.5% and 97.17 ± 0.34% of total cluster passes the filter. 

The raw paired-reads from Miseq were processed using QIIME2. Briefly, reads were trimmed to remove bases with Phred score lower than 30 and quality-filtered with parameters set as previously optimized [29]. Open reference operational taxonomic unit (OTU)-picking was performed, and taxonomical assignment to the generated OTUs was performed with 97% identity against Greengenes database 13.8. Alpha diversity and unweighted principal coordinate analyses plots using the phylogenic tree-based unifrac distance metric were generated using scripts from QIIME package. Bacteria were classified on the basis of the short-chain fatty acids (SCFAs) end-product, as previously described [30,31]. 

### 2.11. Reagents

All reagents were purchased from Merck (Barcelona, Spain), unless otherwise stated. 

### 2.12. Statistical Analysis

The Shannon, Chao, Pielou, and whole observed species were calculated using PAST 4.02. Reads in each OTU were normalized to total reads in each sample. Only taxa with a percentage of reads >0.001% were used for the analysis. Linear discriminant analysis (LDA) scores greater than 2 were displayed. Taxonomy was summarized at the genus level within QIIME2 and uploaded to the Galaxy platform [32] to generate LEfSe/cladogram enrichment plots considering significant enrichment at a *p* < 0.05, LDA score >2. Results are expressed as means ± standard error of the mean (SEM) of measurements. The evolution of tail SBP with time was compared using the nested design, with treatment and days as fixed factors and the rat as a random factor. When the overall difference was significant, we made comparisons using Bonferroni´s method with an appropriate error. Analysis of the nested design was also carried out with groups and concentrations to compare the concentration–response curves to acetylcholine. The remaining variables were tested on normal distribution using the Shapiro–Wilk normality test and compared using a one-way ANOVA and Tukey’s post hoc test in cases of normal distribution, or Mann–Whitney test or Kruskal–Wallis with Dunn’s multiple comparison test in cases of abnormal distribution. *p* < 0.05 was considered statistically significant. 

## 3. Results

### 3.1. Mycophenolate (MMF) Treatment Reduced Gut Dysbiosis in DOCA-Salt Rats

**Figure 1:**
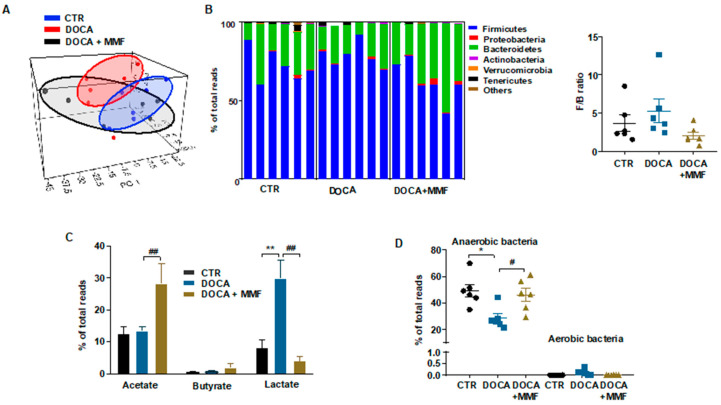
Mycophenolate mofetil (MMF) induced different behaviors of the gut microbiota in deoxycorticosterone acetate (DOCA)-salt rats. The microbial DNA from fecal samples was analyzed by 16S ribosomal ribonucleic acid (rRNA) gene sequencing. (**A**) Principal component analysis in the gut microbiota from all experimental groups. (**B**) Phylum breakdown of the six most abundant bacterial communities in the fecal samples was obtained from all experimental groups and Firmicutes/Bacteroidetes ratio (F/B ratio) was calculated as a biomarker of gut dysbiosis. (**C**) Relative proportion of lactate-, butyrate-, and acetate-producing bacteria expressed as relative proportions of total bacteria. (**D**) Relative proportion of anaerobic and aerobic bacteria in the gut microbiota in control (CTR), untreated DOCA-salt, and DOCA-salt treated with mycophenolate mofetil (DOCA-MMF) rats; *n =* 6. Values are represented as means ± standard error of the mean. * *p* < 0.05 and ** *p* < 0.01 are significant differences compared with CTR. # *p* < 0.05 and ## *p* < 0.01 are significant differences compared with untreated DOCA-salt.

The composition of bacterial communities was analyzed by calculating four key ecological parameters, including Chao richness, Pielou evenness, Simpsons diversity, and the number of observed species. In the DOCA group, Pielou evenness was found to be reduced as compared to control rats without any effect of MMF treatment, whereas it reduced richness and the number of observed species (Figure S1). When the axonometric PCA of the bacterial community was represented, a clear separation between control and DOCA cluster was found. The key bacterial population that is responsible for discriminating among groups was the genus *Lactobacillus* (loading 0.9). The Kaiser–Meyer–Olin (KMO) test was 0.77, indicating a middling sampling for PCA at the genus level. The Barlett’s test of sphericity was <0.05, demonstrating a valid data matrix to continue with the analysis. However, the cluster belonging to the DOCA-MMF group was found intermixed between the two other clusters (Figure 1A). No changes between DOCA-salt and CTR groups were found at the phylum level. MMF treatment was able to promote a reduction in the Firmicutes phylum and increase the levels of the phylum Bacteroidetes compared to DOCA-salt (Table 1, Figure 1B). In agreement with previous data [8,9], the Firmicutes/Bacteroidetes ratio (F/B) ratio, which has been described as an indicator of intestinal dysbiosis [1], was without change in the DOCA-salt model (Figure 1B). Feces from DOCA-salt rats showed a significant increase in the proportion of lactate-producing bacteria without change in acetate- and butyrate-producing bacteria, as compared to the CTR group (Figure 1C). MMF was able to restore the elevation in lactate-producing bacteria found in feces from DOCA-salt joined to an increase in the acetate-producing bacteria (Figure 1C). Furthermore, the populations of strict anaerobic bacteria were significantly depleted in the DOCA-salt group (Figure 1D) in comparison with control group, but no significant differences in strict aerobic bacteria were found. The MMF treatment restored this change in the percentage of anaerobic bacteria in DOCA-salt rats.

**Figure 2:**
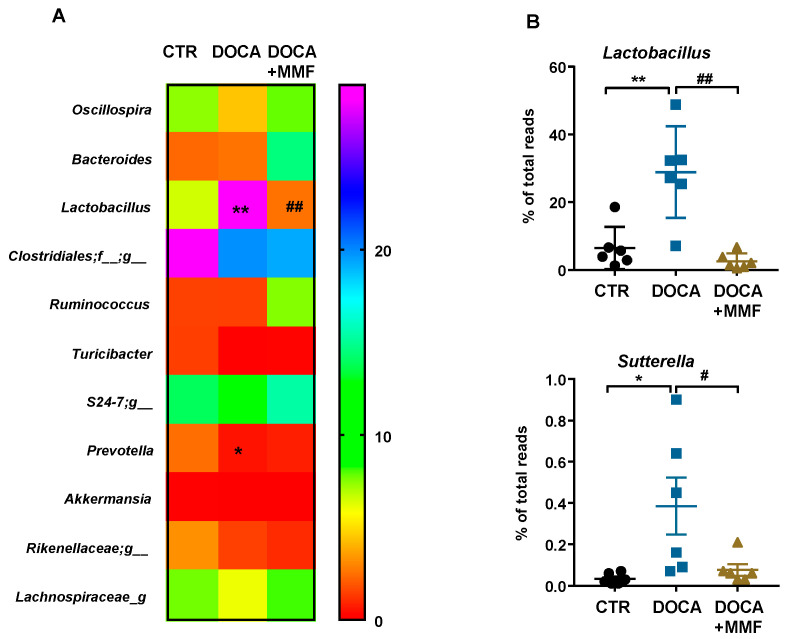
Mycophenolate mofetil (MMF) induced genera changes in the gut microbiota composition in deoxycorticosterone acetate (DOCA)-salt rats. (**A**) The heatmap colors represent the relative percentage of microbial genus assigned within each sample. (**B**) The relative abundance of *Lactobacillus* and *Sutterella* genera. Values are expressed as mean ± SEM (*n =* 6). * *p* < 0.05 and ** *p* < 0.01 compared with control (CTR) group. # *p* < 0.05 and ## *p* < 0.01 are significant differences compared with the untreated DOCA-salt group.

Figure S2 shows the alteration in bacterial taxa (class, order, family, and genus) observed in DOCA-salt rats through LDA. In the DOCA-salt group, 25 taxa were increased (green) and 20 were decreased (red), as compared to the CTR group (Figure S2A). The MMF-treated DOCA-salt group showed a great number of changes; in particular, 21 taxa were increased (red) and 21 were decreased (green), as compared to the DOCA group (Figure S2B). The heat map shows an analysis of the relative expression of the 11 major genera found in DOCA-salt rats (Figure 2A,B). Several authors have described the ability of certain bacteria to modulate the immune system, such as *Lactobacillus, Bifidobacterium,* and *Sutterella* [33,34], among others. We found higher amounts of the genus *Lactobacillus* (from the Lactobacillaceae family) in hypertensive rats, with MMF being able to normalize them (Figure 2B). In addition, the genus *Sutterella*, which at low levels within the gut microbiome is associated with gut immune homeostasis [34], was found elevated in the DOCA-salt group, and MMF induced a contraction in this genus (Figure 2B).

### 3.2. Mycophenolate (MMF) Improved Intestinal Integrity, α-Defensin Expression, and Changed MLN T Cell Populations in DOCA-Salt Rats

**Figure 3:**
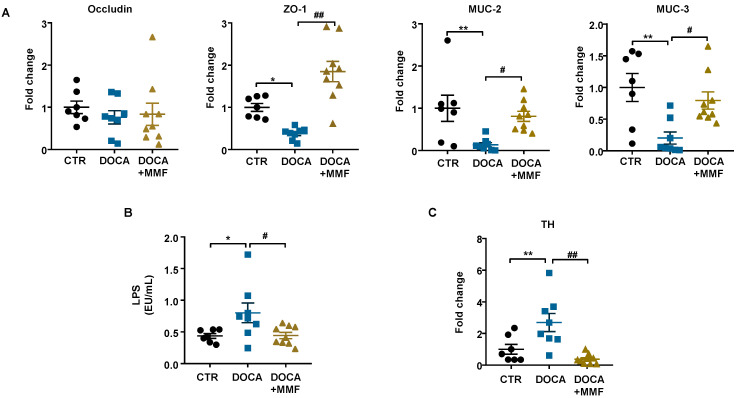
Mycophenolate mofetil (MMF) induced improvement of gut integrity and sympathetic tone in the gut in deoxycorticosterone acetate (DOCA)-salt rats. (**A**) Colonic mRNA levels of occludin, zonula occludens-1 (ZO-1), mucin (MUC)-2, and MUC-3. (**B**) Plasma levels of lipopolysaccharide (LPS). (**C**) Colonic mRNA levels of tyrosine hydroxylase (TH). Values are expressed as mean ± SEM (*n =* 7–9). * *p* < 0.05 and ** *p* < 0.01 compared with control (CTR) group. # *p* < 0.05 and ## *p* < 0.01 are significant differences compared with the untreated DOCA-salt group.

Hypertension is linked to a decrease in the expression of gut tight junction proteins, together with a raise in permeability, and gut pathology [35,36]. We observed similar mRNA levels of barrier-forming junction protein ocludin and lower levels of zonula occludens-1 (ZO-1) in colonic samples from the DOCA-salt group in comparison with control rats (Figure 3A). MMF increased ZO-1 expression levels in the colon, suggesting an improvement in the barrier function. Increased gut permeability has also been associated with low numbers of goblet cells [15]. Goblet cells excrete mucins, protecting the gut from pathogen invasion, and thus regulating the gut immune response [37]. We have found a downregulation of mucin (MUC)-2 and MUC-3 transcripts in hypertensive DOCA-salt rats, which was suppressed by the MMF treatment (Figure 3A). These results point to an increase in intestinal permeability in DOCA-salt that would allow bacterial components (e.g., LPS) to enter the bloodstream. We quantified plasma endotoxin levels and found them significantly higher in the DOCA-salt group in comparison with the control group (Figure 3B). Interestingly, the long-term treatment with MMF decreased endotoxemia (Figure 3B). 

Santisteban et al. [15] showed that the increase in the sympathetic nerve activity to the gut leads to alteration of gut junction proteins in SHR. We found an increase in the expression of tyrosine hydroxylase (TH), a crucial enzyme in the generation of noradrenaline, in the gut in the DOCA-salt group compared with the control group, which was abolished by the chronic MMF treatment (Figure 3C). These results suggest that reduced sympathetic tone in the gut could be associated with the positive effects of MMF in the gut.

Epithelial intestinal cells are able to produce α-defensins, cysteine-rich cationic peptides with antibiotic activity against a broad range of microorganisms [38], in order to keep a stable composition of intestinal microbiota [39]. In DOCA-salt rats, the mRNA levels of rat neutrophil-derived α defensins (RNP)1-2 and RNP4 were increased, whereas RNP3 was decreased as compared with normotensive rats (Figure S3). MMF restored the expression levels of these defensins to levels similar to those found in control groups, and reduced RNP5.

### 3.3. Mycophenolate (MMF) Improved the T Cell Profile at Mesenteric Lymph Nodes (MLNs) in DOCA-Salt Rats

**Figure 4:**
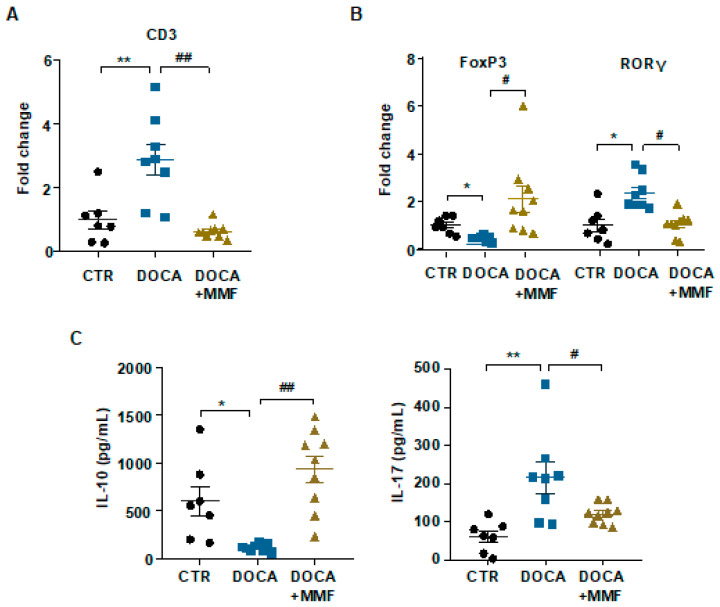
Mycophenolate (MMF) improved the T cell profile of mesenteric lymph nodes (MLNs) in deoxycorticosterone acetate (DOCA)-salt rats. mRNA levels of cluster of differentiation (CD)3 (**A**), forkhead box P3 (FoxP3), and retinoid-related orphan receptor-γ (RORγ) (**B**) in MLNs. (**C**) Concentration of interleukin (IL)-17a and IL-10 was measured by ELISA in lymphocyte-conditioned medium obtained with MLNs from all experimental groups. Values are expressed as mean ± SEM (*n =* 7–9). * *p* < 0.05 and ** *p* < 0.01 compared with control (CTR) group. # *p* < 0.05 and ## *p* < 0.01 are significant differences compared with the untreated DOCA-salt group.

As already explained, bacteria can translocate through the intestinal barrier, leading to the activation of machrophages and dendritic cells and their migration to draining lymph nodes of the lower intestinal tract when under altered gut mucosal integrity [40]. These CX3CR1+ cells can also present soluble antigens to naïve cluster of differentiation (CD)4+ T cells, causing T cell activation. In our experiments, the number of total lymphocytes in MLNs was higher in DOCA-salt rats, measured by RT-PCR, in comparison with the control group. The MMF treatment normalized the content in lymphocytes in MLNs (Figure 4A). The percentage of regulatory T cells (Tregs) was reduced in the DOCA-salt group, and became normalized by MMF treatment. The percentage of Th17 lymphocytes was increased in MLNs from the DOCA-salt group in comparison with control. MMF was able to reduce Th17 populations in MLNs from the DOCA group (Figure 4B). The level of cytokines produced by Treg (IL10) was found to be reduced in lymphocyte-conditioned medium obtained with MLNs from the DOCA-salt group, being elevated by the MMF treatment. The level of IL17, which is mainly produced by Th17, was higher in the DOCA-salt group, and MMF normalized it (Figure 4C).

### 3.4. Mycophenolate Mofetil (MMF) Reduced BP, Improves the Vascular Nitric Oxide (NO) Pathway, and Reduced Oxidative Stress and Inflammation in Aorta and Brain

**Figure 5:**
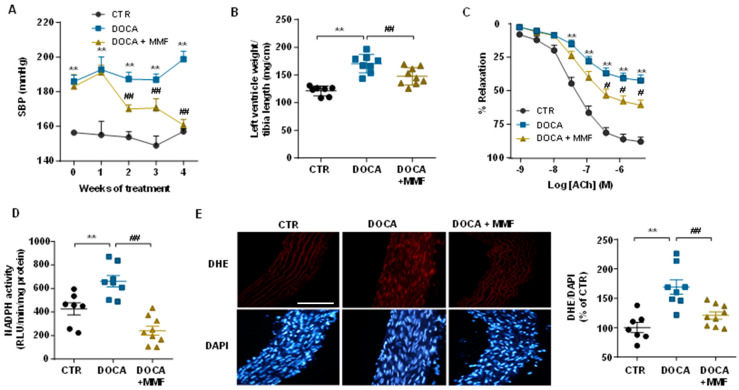
Mycophenolate mofetil (MMF) reduced blood pressure and improved aortic endothelial function in deoxycorticosterone acetate (DOCA)-salt rats. (**A**) Time course of systolic blood pressure (SBP) measured by tail-cuff plethysmography. (**B**) Ratio of left ventricle/tibia length (mg/cm). (**C**) Endothelium-dependent relaxation induced by acetylcholine (ACh) in aortas precontracted by phenylephrine. Nicotinamide adenine dinucleotide phosphate (NADPH) oxidase activity (**D**) and in situ intracellular reactive oxygen species (ROS) (**E**) in aorta from all experimental groups. Values are expressed as mean ± SEM (*n =* 7–9). Scale bar (100 μm). ** *p* < 0.01 is significant differences compared with control (CTR). ## *p* < 0.01 is significant differences compared with untreated DOCA-salt group.

As was described previously [18], chronic MMF treatment showed a progressive decrease in SBP (Figure 5A), which was significant after 2 weeks of treatment, reaching a total reduction of 22.3 ± 3.4 mmHg in the DOCA-salt group. MMF induced a significant reduction of left ventricular hypertrophy (≈13%) in the DOCA-salt group (Figure 5B). Aortae from hypertensive rats showed strongly reduced endothelium-dependent vasodilator responses to acetylcholine compared to aortas from the control groups, for which responses were improved after MMF treatment (Figure 5C). The incubation with L-NAME in the organ bath abolished the normal relaxation induced by acetylcholine in all experimental groups, proving NO to be involved in this relaxation (data not shown). In addition, no differences were observed among groups in the endothelium-independent vasodilator responses to the NO donor sodium nitroprusside in aortic rings, excluding changes in the NO pathway in smooth muscle (data not shown). The presence of VAS2870 in the organ bath increased the relaxation response to acetylcholine in the untreated DOCA-salt group until reaching similar relaxation percentages to those found in control groups (data not shown), suggesting that an increased NADPH oxidase activity is involved, at least in part, in the impaired relaxation to acetylcholine in the aorta in the DOCA-salt group. In fact, NADPH oxidase activity was found to be reduced in the group treated with MMF in comparison with the DOCA-salt group (Figure 5D). In addition, rings from DOCA-salt showed marked increased ROS content (≈70%), measured by red staining to ethidium in the vascular wall, which was reduced by MMF treatment (Figure 5E).

**Figure 6:**
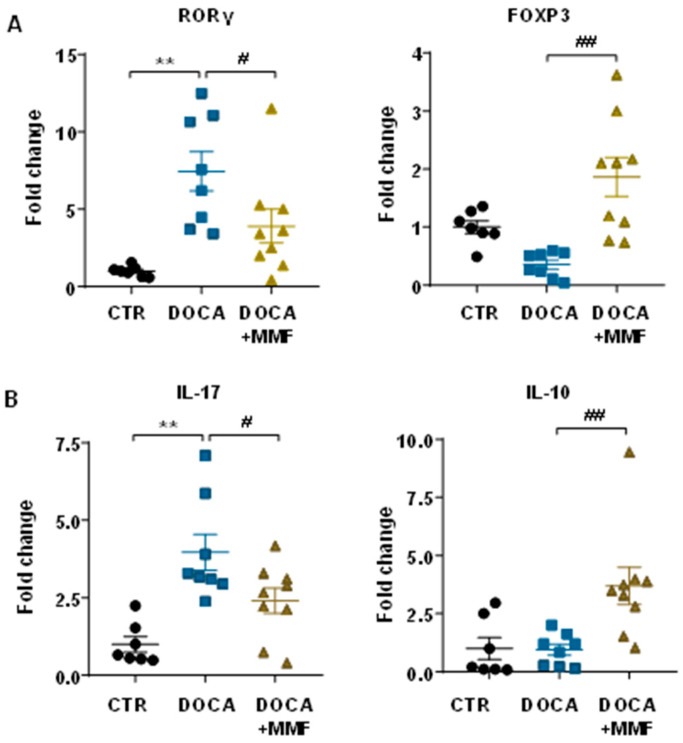
Mycophenolate mofetil (MMF) induced changes in T cell infiltration in the vascular wall from deoxycorticosterone acetate (DOCA)-salt rats. (**A**) T cell infiltration in aortas from all experimental groups was measured by mRNA levels of T helper (Th)17 (RORγ) and regulatory T cells (FoxP3). (**B**) Cytokine content analyzed by mRNA levels of IL17a and IL10 in aorta from all experimental groups. Values are expressed as mean ± SEM. ** *p* < 0.01 is significant differences compared with control (CTR). # *p* < 0.05 and ## *p* < 0.01 are significant differences compared with untreated DOCA-salt rats.

The infiltration of T cells was analyzed measuring the mRNA levels of transcription factor FoxP3 and retinoid-related orphan receptor-γ (RORγ) as markers of accumulation of Treg and Th17, respectively, in aorta from all experimental groups (Figure 6A). Increased Th17 cell infiltration was shown in aorta from hypertensive rats, whereas non significative change in Treg content was detected in aorta from the DOCA-salt group. The MMF treatment reduced Th17 cells and increased Treg cell infiltration in aorta from DOCA-salt rats (Figure 6A). Interestingly, linked to T cells infiltration, the mRNA levels of the proinfalmmatory cytokine IL-17a were higher in DOCA-salt rats as compared to control group (Figure 6B). Again, the treatment with MMF restored the mRNA levels of IL-17a, whereas increased IL-10 content in aortic homogenates from DOCA-salt rats.

**Figure 7:**
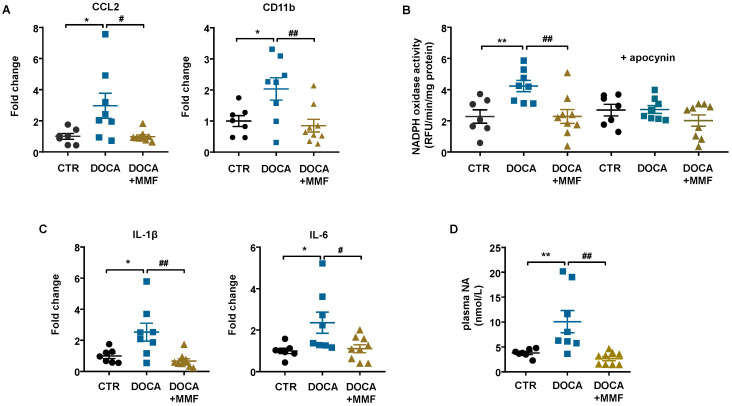
Mycophenolate mofetil (MMF) reduced immune cell infiltration, neuroinflammation, and NADPH oxidase activity in the paraventricular nucleus (PVN) of the hypothalamus in deoxycorticosterone acetate (DOCA)-salt rats. (**A**) mRNA levels of C-C motif chemokine ligand 2 (CCL2) and cluster of differentiation molecule 11B (CD11b). (**B**) NADPH oxidase activity measured by dihydroethidium (DHE) fluorescence measured in the microplate reader in homogenates from brain PVN in the absence and presence of the non-selective NADPH oxidase inhibitor apocynin (50 μM). (**C**) mRNA levels of pro-inflammatory cytokines interleukin (IL)-1β and IL-6 and homogenates from brain PVN. (**D**) Plasma levels of noradrenaline (NA) expressed in nmol/L. Values are expressed as mean ± SEM (*n =* 7–9). * *p* < 0.05 and ** *p* < 0.01 are significant differences compared with control (CTR). # *p* < 0.05 and ## *p* < 0.01 are significant differences compared with untreated DOCA-salt rats.

The PVN of the hypothalamus has been shown to play an important role in the development of this form of hypertension, one that involves the sympathetic nervous system [41]. Brain neuroinflammation has been described after 3 weeks of DOCA-salt administration [42]. We found that mRNA levels of C-C chemokine ligand 2 (CCL2) and macrophage marker CD11b were increased in brain PVN from hypertensive rats, which were normalized by MMF (Figure 7A). The NADPH oxidase activity (Figure 7B) and the mRNA levels of pro-inflammatory cytokines (IL-1β and IL-6) (Figure 7C) were also increased in the PVN of the DOCA-salt group and decreased by MMF treatment. The plasma NA levels were increased in DOCA-salt rats and normalized by MMF (Figure 7D). 

## 4. Discussion

The main findings of this study are the following: (1) chronic MMF treatment reduced gut dysbiosis in hypertensive DOCA-salt rats; (2) shifts in gut microbiota composition induced by MMF were linked to an improvement in gut integrity and the normalization of colonic α-defensins production; and (3) MMF reduced brain PVN NADPH oxidase activity, neuroinflammation, and sympathetic activity. 

Abundant evidence has demonstrated the association between gut dysbiosis, the immune system, and hypertension [1,3,4,11,43]. Our results are consistent with data previously described, with MMF being able to prevent BP increase [17,18] and improve aortic endothelial dysfunction, increasing NO availability in DOCA-salt animals [18]. Furthermore, we found that the MMF treatment induced a modulation in the aortic immune cell infiltration, inducing a reduction in the pro-inflammatory and pro-oxidative cytokine profile.

Several studies have described the ability of immunomodulatory drugs to modulate the gut microbiota, inducing dysbiosis [44] or improving the dysbiotic condition found in several pathologies [45]. Last year, the way in which gut dysbiosis is displayed in DOCA-salt hypertension was reported [8,9]. Our results are in agreement with the key known characteristics of gut microbiota described in DOCA-salt animals [8,9], such as a reduced evenness, no change in the F/B ratio, and a higher proportion of lactate-producing bacteria. MMF treatment induced a remodeling in the gut microbiota, normalizing the proportion of bacteria belonging to Firmicutes and Bacteroidetes and reducing lactate-producing bacteria. In addition, MMF increased acetate-producing bacteria, which could contribute to reduce BP. In fact, the increase in acetate-producing bacteria induced by high-fiber diet, *Bifidobacterium breve* consumption, or acetate supplementation were associated with decreased BP, improvement of vascular endothelial and cardiac dysfunction, and attenuation of cardiac and renal fibrosis in DOCA-salt animals [8,9]. 

BP-lowering effects of MMF have been associated with decreased circulating and renal T cells in SHR and DOCA-salt rats, although MMF reduced both T cell subtypes, Th17 and Tregs [17,18,46]. However, we found increased Tregs and IL-10 (the main cytokine produced by Treg) in both MLNs and aortas of MMF-treated rats, which could contribute to their antihypertensive effects, since IL-10 released by Tregs improves endothelial function and reduces BP in hypertensive mice [28]. Several authors have described the ability of certain SCFAs, such as acetate and butyrate, to modulate the immune system. Concretely, acetate is able to induce an elevation in Treg populations [9,47]. We found an expansion in acetate-producing bacteria induced by MMF treatment in DOCA-salt rats, which could be involved in the higher numbers of Treg and IL-10 found in MLN and aorta from the DOCA-MMF group. Otherwise, in DOCA-salt rats, we found an elevation in the genus *Sutterella*, which has been described to elevate Th17 populations [48]. MMF reduced *Sutterrella* contents in feces from DOCA-salt rats, possibly leading to lower Th17. This effect in the microbiota could increase the direct inhibitory effect of MPA, the active form of MMF, inhibiting IL-17 expression [49]. In addition, the increased abundance of *Lactobacillus* spp. found in DOCA samples was also normalized by the MMF treatment, similarly to acetate consumption [9]. This is an important beneficial effect because *Lactobacillus* spp. has been shown to elevate certain pro-inflammatory cytokines such as IL-6, tumor necrosis factor (TNF)α, or interferon (INF)γ in enterocytes [50,51]. 

Multiple possibilities wherein MMF might elicit changes in gut microbiota were found. It has been repeatedly shown how a change in the host health status is accompanied by changes in the composition of gut microbiota. Therefore, microbiota could be adapted to BP reduction, shifting to a microbiota composition similar to normotensive rats. However, we previously demonstrated that hydralazine, which normalized BP in SHR, was unable to improve dysbiosis [6], ruling out the hypothesis that gut microbiota are adapted to normotensive conditions. Changes in gut microbiota composition have been associated with gut integrity [2,6]. The mammalian digestive tract epithelial cells create a tight barrier in the gut, contributing to the hypoxic environment of the lumen. Damage to this barrier makes the environment less hypoxic, conducive to aerobic bacterial growth [52,53]. We found a significant reduction in the mRNA expression of tight junction protein ZO-1 and mucins in colon from DOCA-salt rats, suggesting reduced colonic integrity and increased gut permeability in hypertensive rats. The possible impairment of gut barrier function was supported by the translocation of endotoxin from the intestinal lumen to the bloodstream, leading to higher LPS plasma levels in the DOCA-salt group. MMF treatment increased colonic ZO-1 expression and normalized MUC-2 and MUC-3 mRNA levels, suggesting improvement of gut barrier function. In fact, MMF inhibited the LPS translocation to the systemic circulation. In addition, intestines of angiotensin II-hypertensive mice and SHR were significantly less hypoxic and with increased aerobic bacteria in feces, due to a reduction in the epithelium barrier integrity [2,6]. We also found decreased abundance of anaerobic bacteria in feces from DOCA-salt rats, which was associated with a loss of gut integrity. DOCA-salt rats treated with MMF showed increased colonic integrity and a proportion of strict anaerobic bacteria similar to control groups. These data reinforce the key role of gut integrity in the composition of intestinal microbiota. In addition, intestinal epithelial cells and Paneth cells secrete antimicrobial peptides, such as defensins, which selectively kill Gram-positive bacteria [54,55,56,57]. Components of the microbiota, such as LPS, are recognized by Toll-like receptors expressed by these intestinal cells and trigger production and secretion of these defensins. We found changes in the expression levels of defensins in colonic samples from the DOCA group in comparison with control group, which might also be involved in changes in microbiota found in hypertensive rats. MMF restored defensin expression to become similar to that found in normotensive rats. Overall, MMF treatment, by increasing acetate-producing bacteria and possibly acetate content in feces, might increase gut barrier function, reducing endotoxemia and improving Th17/Treg balance in MLNs, reducing Th17 infiltration in vascular tissues, which participate in reducing BP. However, whether the remodeling induced by MMF in gut microbiota contributes to lower BP in DOCA-salt rats requires further investigation using fecal microbiota transplantation from the donor DOCA-MMF group to recipient hypertensive DOCA-salt rats.

Our current study is consistent with the work of Santisteban et al. [15], demonstrating an increased gut sympathetic drive (increased TH expression) linked to the loss of gut integrity and microbial dysbiosis in hypertensive animals. Moreover, Robles-Vera et al. [6] demonstrated that reduction of sympathetic activity in the colon induced by losartan improved gut integrity and increased anaerobic bacteria, whereas hydralazine (that increased gut sympathetic drive) was unable to restore gut integrity and microbiota composition. We also found increased mRNA levels of TH in the colon from DOCA-salt rats, which were normalized by MMF treatment. In recent years, the hypothesis that establishes the presence of a brain–gut communication driven by the sympathetic system has gained prevalence. Central administration of a modified tetracycline inhibited microglial activation, normalized sympathetic activity, attenuated pathological alterations in the gut wall, restored certain gut microbial communities altered by angiotensin II, and reduced BP [58]. Santisteban et al. [15] observed enhanced gut–neuronal communication in hypertension originating from the PVN of the hypothalamus and presenting as increased sympathetic drive to the gut. In the brain, the NADPH oxidase-dependent ROS production activates the sympathetic outflow [59]. In brain PVN from the DOCA-salt group, we found an increase in NADPH oxidase activity-driven ROS production and higher expression of pro-inflammatory cytokines (IL-1β and IL-6) than in control rats, which was associated with increased plasma NA levels. The increased sympathetic activity also affects the bone marrow (BM), resulting in an increase in inflammatory cells, which migrate to the PVN and enhance neuroinflammation [35]. Accordingly, we found increased inflammation in brain PVN from DOCA-salt rats, associated with an increased expression of CCL2, which facilitates BM cells entering the brain’s parenchymal space, and CD11b, a macrophage marker that might contribute to neuroinflammation. MMF reduced immune cells infiltration, neuroinflammation, and NADPH oxidase activity in brain areas of cardiovascular control such as PVN, and reduced plasma NA content. Our results are in agreement with the reduced brain macrophage/glial activation induced by MMF in stroke-prone SHR [60]. The brain changes induced by MMF in DOCA-salt rats might reduce the sympathetic excitation (lower plasma NA level), lending to lower gut sympathetic drive (lower colonic TH expression). The gut sympathetic tone is a crucial modulator of gut barrier integrity and microbiota composition [6,27]. Our results also support this hypothesis because MMF, which reduced colonic TH in DOCA-salt rats, improved gut integrity and the gut dysbiosis. 

## 5. Conclusions

We found for the first time that MMF induces remodeling of gut microbiota in DOCA-salt rats. This seems to be linked to its capacity to improve gut integrity due to reduced sympathetic drive in the gut associated with the reduced brain neuroinflammation. 

## Figures and Tables

**Table 1:** Effects of mofetil mycophenolate (MMF) in deoxycorticosterone acetate (DOCA)-salt rats on phyla changes in the gut microbiota.

Phylum	CTR (n = 6)	DOCA (n = 6)	DOCA+MMF (n = 6)
Firmicutes	72.6 ± 4.8	78.6 ± 3.5	62.4 ± 5.7 #
Proteobacteria	0.4 ± 0.3	0.7 ± 0.3	1.3 ± 0.7
Bacteroidetes	25.0 ± 4.4	19.0 ± 3.4	35.2 ± 5.6 #
Actinobacteria	0.1 ± 0.0	0.4 ± 0.1	0.3 ± 0.1
Verrucomicrobia	0.1 ± 0.1	0.0 ± 0.0	0.0 ± 0.0
Tenericutes	1.5 ± 0.7	0.7 ± 0.4	0.2 ± 0.1
Others	2.8 ± 0.1	0.2 ± 0.0	0.3 ± 0.2

Values are expressed as mean ± SEM. Control rats (CTR). # *p* < 0.05 compared with the DOCA group.

## Supplementary Materials

The following are available online at https://www.mdpi.com/2076-3921/9/12/1199/s1: Figure S1: Effects of mycophenolate mofetil (MMF) in ecological parameters of the gut microbiota from deoxycorticosterone acetate (DOCA)-salt rats. Figure S2: Distinct changes in the gut microbiota between control (CTR) and deoxycorticosterone acetate (DOCA)-salt rats and mycophenolate mofetil (MMF) treatment. Figure S3: Mycophenolate mofetil (MMF) induced improvement of α-defensin expression in deoxycorticosterone acetate (DOCA)-salt rats. Table S1: Primers for real-time RT-PCR.
