# Supplementary material for: Mycophenolate Improves Brain–Gut Axis Inducing Remodeling of Gut Microbiota in DOCA-Salt Hypertensive Rats
Source: Antioxidants (Basel). 2020 Nov 28;9(12):1199. doi: 10.3390/antiox9121199 (PMC7761232; doi:10.3390/antiox9121199)
Supplement: Supplementary file 1 [file antioxidants-09-01199-s001.pdf]

Table S1. Primers for real-time RT-PCR.

mRNA targets	Descriptions (Gene ID)	Forward	Reverse
IL-6	interleukin-6 (24498)	GATGGATGCTTCCAAACTGG	AGGAGAGCATTGGAAGTTGG
IL-10	interleukin-10 (25325)	GAATTCCCTGGGAGAGAAGC	GCTCCACTGCCTTGCTTTTA
IL-17a	interleukin-17a (301289)	CTTCACCTTGGACTCTGAGC	TGGCGGACAATAGAGGAAAC
IL1β	Interleukin-1β (24494)	GTCACTCATTGTGGCTGTGG	GCAGTGCAGCTGTCTAATGG
CCL2	C-C Motif Chemokine Ligand 2 (24770)	CCTCCACCACTATGCAGGTC	CAGCCGACTCATTGGGATCA
CD3	Cluster of differentiation 3 (25710)	CGTCCGCCATCTTGGTAGAGAGAGCAT	CTACTGCTGTCAGGTCCACCTCCAC
CD11b	Cluster of differentiation 11b (25021)	GAGAACTGGTTCTGGCTTGC	TCAGTTCGAGCCTTCTT
FoxP3	forkhead box P3 (317382)	AGGCACTTCTCCAGGACAGA	CTGGACACCCATTCCAGACT
RORϒ	ROR-gamma 1(9885)	GCCTACAATGCCAACAACCACACA	TGATGAGAACCAAGGCCGTGTAGA
Occludin	Occludin (83497)	AGCCTGGGCAGTCGGGTTGA	ACACAGACCCCAGAGCGGCA
Muc2	mucin-2 (24572)	CGATCACCACCATTGCCACTG	ACCACCATTACCACCACCTCAG
Muc3	mucin-3 (687030)	CACAAAGGCAAGAGTCCAGA	AGTGTCCTTGGTGCTGCTGAATG
ZO-1	zonula occludens-1 (292994)	GCCAGCCAGTTCCGCCTCTG	AGGGTCCCGGGTTGGTG
Th	tyrosine hydroxylase (25085)	GATTGCTACCTGGAAGGAGGT	AGTCCAATGTCCTGGGAGAAC
RNP1-2	alpha defensin RNP1-2 (613220)	GGACGCTCACTCTGCTTACC	TGGATTCTTCTTGGTCGGAG
RNP3	alpha defensin RNP3 (498659)	AAGAGCGCTGTGTCTCTTGC	CAACAGAGTCGGTAGATGCG
RNP4	alpha defensin RNP4 (286958)	TCTGCTCATCACCCTTCTCC	AACAGAGACGGTAGATGCGG
RNP5	alpha defensin RNP5 (28699)	ACCAGGCTTCAGTCATGAGG	CATCCCATTGGTTCTTGGTC
GAPDH	glyceraldehyde-3-phosphate dehydrogenase (28383)	GTCGGTGTGAACGGATTT	ATGGGTTTCCCGTTGATG

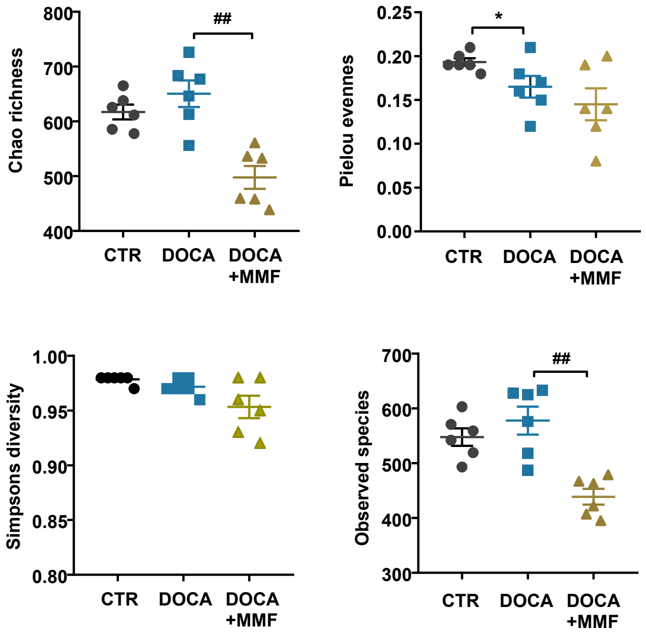

Figure S1. Effects of Mycophenolate mofetil (MMF) in ecological parameters of the gut microbiota from Deoxycorticosterone acetate (DOCA)-salt rats. The microbial DNA from faecal samples was analyzed by 16S rRNA gene sequencing. To evaluate general differences of microbial composition amongst all experimental groups, richness, evenness, diversity, and observed species were examined in feces. n = 6 rats per experimental group in each comparison. *P < 0.05 significant differences compared with Control (CTR*)*. ##P < 0.01 significant differences compared with untreated DOCA**.**

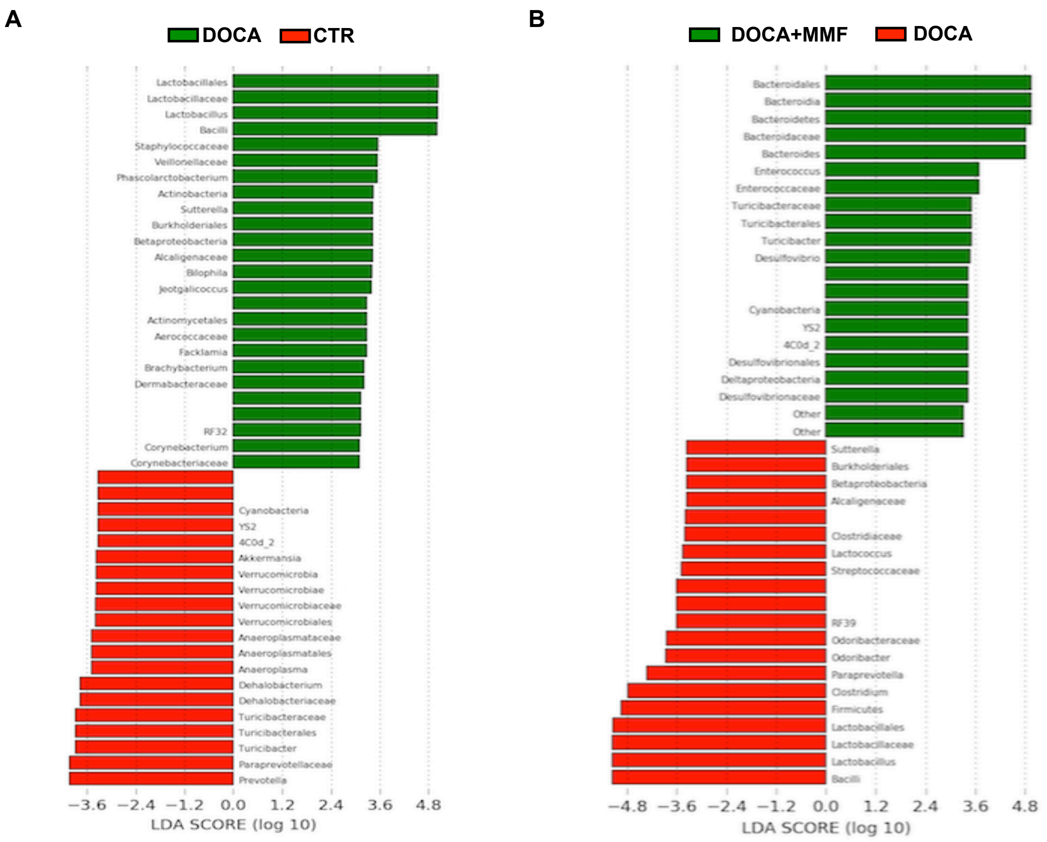

Figure S2. Distinct changes in the gut microbiota between Control (CTR) and Deoxycorticosterone acetate (DOCA)-salt rats and Mycophenolate mofetil (MMF) treatment. A) Comparisons of microbiome changes in CTR *versus* DOCA-salt. (red bars represent CTR-enriched taxa, green bars represent DOCA-salt-enriched taxa). B) Comparisons of microbiome changes in DOCA-salt *versus* DOCA-MMF (green bars represent DOCA-MMF-enriched taxa, red bars represent DOCA-enriched taxa). Linear discriminant analysis effect size (LEfSe) identified significantly different bacterial taxa enriched in each cohort at LDA Score > 2, P < 0.05. N = 6 rats per experimental group in each comparison.

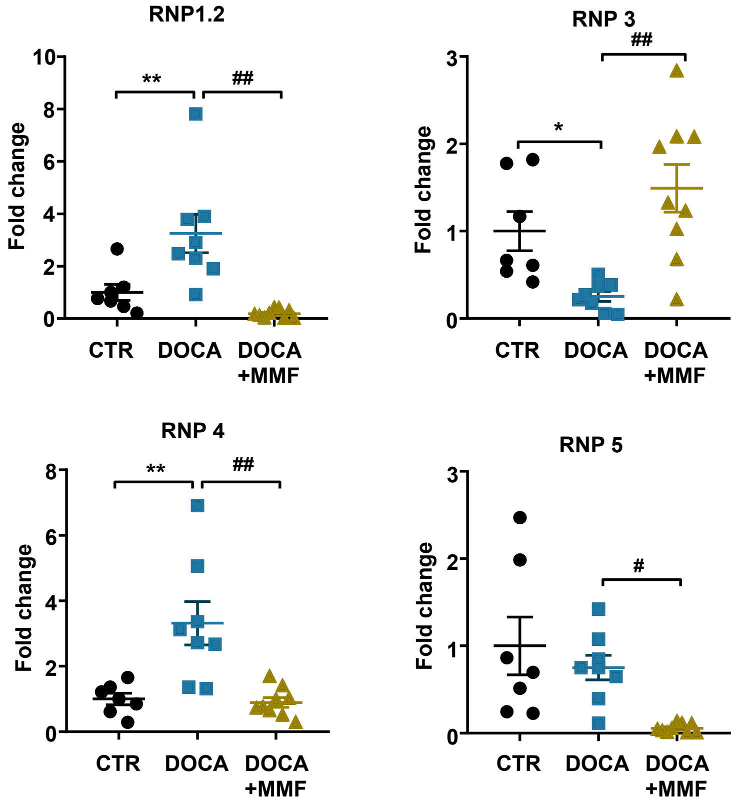

Figure S3. Mycophenolate mofetil (MMF) induces improvement of α-defensins expression in Deoxycorticosterone acetate (DOCA)-salt rats. mRNA levels of α-defensins (RNP1.2, RNP3, RNP4, and RNP5) in colon from untreated Wistar (CTR), untreated DOCA-salt or DOCA treated with MMF (DOCA+MMF). Values are expressed as mean ± SEM. *P < 0.05 and **P < 0.01 compared with Control (CTR). #P < 0.05 and ##P < 0.01 compared with the untreated DOCA.
